# Integrating whole transcriptome sequence data and public databases for analysis of somatic mutations in tumors

**DOI:** 10.1186/gb-2011-12-s1-p44

**Published:** 2011-09-19

**Authors:** Amin A Momin, Brian P James, Thomas C Motter, Humam N Kadara, Garth Powis, Ignacio I Wistuba

**Affiliations:** 1 Department of Bioinformatics and Computational Biology, The University of Texas MD Anderson Cancer Center, Houston, TX 77030, USA; 2 Department of Experimental Therapeutics, The University of Texas MD Anderson Cancer Center, Houston, TX 77030, USA; 3 Department of Thoracic/Head and Neck Thoracic Oncology, The University of Texas MD Anderson Cancer Center, Houston, TX 77030, USA; 4 Department of Pathology, The University of Texas MD Anderson Cancer Center, Houston, TX 77030, USA

## 

The annotation of pathologically relevant somatic variations has gained importance with the wide use of next-generation sequencing in biomedical studies. At present, this evaluation is performed using public tools such as SAMtools and ANNOVAR by comparing predicted mutations and small nucleotide variations (SNVs) with databases such as 1000 Genomes and dbSNP, as well as with paired normal data if available. However, these analytical methods lack the ability to integrate information from the different analyses into a single output. Additionally, many approaches are filter based and remove data that does not match specific criteria, thus leading to the removal of variations that would otherwise be reconsidered later. To this end, we have developed a Perl wrapper script that utilizes standard next-generation sequencing output files along with SAMtools and ANNOVAR to produce an annotated tumor variant file with sequence calls from related tumor and matched normal samples.

We performed SOLiD paired-end sequencing of the whole transcriptome of one lung adenocarcinoma and seven normal lung samples (including one matched normal). BioScope 1.3 was used to map the reads, and the SNVs were identified by the diBayes package. The map files in binary-sequence alignment format (BAM) and SNV files in generic feature format (GFF) were used to annotate the tumor SNVs with matched normal sequence information at each position (diBayes and SAMtools), as well as other normal samples (both position and gene based). Furthermore, SNVs were annotated with positional information, including whether intronic, exonic, or synonymous versus nonsynonymous, as well as with data from the 1000 Genomes Project (allele frequency), the dbSNP database (rs identifiers) and the Catalogue of Somatic Mutations in Cancer (COSMIC) database. Of the 1,804 SNVs initially identified in the tumor sample, 138 SNVs were found in non-coding RNA, and 75 did not appear in the normal samples according to diBayes or in the specific matched normal sample according to SAMtools. Because the capacity to sequence the whole transcriptome is subject to the expression level, the possibility of failure to detect variations in normal lung samples cannot be ignored. To address this concern, we analyzed 1000 Genomes data and found that only 23 of the 75 potential tumor-specific SNVs exhibited allele frequencies <1%, and 6 of these exist in dbSNP. All of these steps can be rapidly performed by a researcher, and modifying the approach to identify other types of SNVs is easily achievable.

The use of a single script that tracks input file names and locations is expected to improve data handling and reporting. Notably, all variant data are present in a single file, allowing straightforward modification of criteria and instant hypothesis testing and therefore reducing the need for an informed end user to re-engage a bioinformatician to address another biological question.

